# Predictors of long term weight loss maintenance in patients at high risk of type 2 diabetes participating in a lifestyle intervention program in primary health care: The DE-PLAN study

**DOI:** 10.1371/journal.pone.0194589

**Published:** 2018-03-23

**Authors:** Aleksandra Gilis-Januszewska, Noël C. Barengo, Jaana Lindström, Ewa Wójtowicz, Tania Acosta, Jaakko Tuomilehto, Peter E. H. Schwarz, Beata Piwońska-Solska, Zbigniew Szybiński, Adam Windak, Alicja Hubalewska-Dydejczyk

**Affiliations:** 1 Chair and Department of Endocrinology, Jagiellonian University, Medical College, Krakow, Poland; 2 Department of Medical and Population Health Science Research, Herbert Wertheim College of Medicine, Florida International University, Miami, United States of America; 3 Chronic Disease Prevention Unit, National Institute for Health and Welfare (THL), Helsinki, Finland; 4 Department of Public Health, Universidad del Norte, Barranquilla, Colombia; 5 Centre for Vascular Prevention, Danube-University Krems, Krems, Austria; 6 Department of Chronic Disease Prevention, National Institute for Health and Welfare, Helsinki, Finland; 7 Diabetes Research Group, King Abdulaziz University, Jeddah, Saudi Arabia; 8 Dasman Diabetes Institute, Dasman, Kuwait; 9 Department for Prevention & Care of Diabetes, Medical Clinic Unit III, University Clinic Carl Gustav Carus at Technical University Dresden, Dresden, Germany; 10 Paul Langerhans Institute Dresden of the Helmholtz Center Munich at University Hospital and Faculty of Medicine, Technical University Dresden, Dresden, Germany; 11 German Center for Diabetes Research, Neuherberg, Germany; 12 Department of Family Medicine, Chair of Medicine and Gerontology, Jagiellonian University Medical College, Krakow, Poland; International University of Health and Welfare School of Medicine, JAPAN

## Abstract

Lifestyle interventions in type 2 diabetes (DM2) prevention implementation studies can be effective and lasting. Long-term weight loss maintenance enhances the intervention effect through a significant decrease in diabetes incidence over time. Our objective was to identify factors predicting long-term successful weight reduction maintenance achieved during a DM2 prevention program in patients with high DM2 risk in primary health care. Study participants (n = 263), middle-aged, slightly obese with baseline increased DM2 risk (Finnish Diabetes Risk Score (FINDRISC)>14), but no diabetes were invited to receive 11 lifestyle counselling sessions, guided physical activity sessions and motivational support during 10-months. The study participants had three clinical examinations during the study (baseline, one and three years). Stepwise regression analysis was used to determine demographic, clinical, and lifestyle predictors of weight reduction maintenance two years after the discontinuation of the intervention. Out of 105 patients who completed all three examinations (baseline age 56.6 (standard deviation (SD) = 10.7), body mass index 31.1 kg/m^2^ (SD = 4.9), FINDRISC 18.6 (SD = 3.1)), 73 patients (70%) showed weight loss during the intervention (mean weight loss 4.2 kg, SD = 5.1). The total weight loss achieved in the maintainers (27 of 73 study participants) two years after the intervention had finished was 6.54 kg (4.47 kg+2.0 kg). The non-maintainers, on the other hand, returned to their initial weight at the start of the intervention (+0.21 kg). In multivariable analysis baseline history of increased glucose (odds ratio (OR) = 3.7; 95% confidence interval (CI) 1.0–13.6) and reduction of total fat in diet during follow-up (OR = 4.3; 95% CI 1.5–12.2) were independent predictors of successful weight loss. Further studies exploring predictors of weight loss maintenance in diabetes prevention are needed to help health care providers to redesign interventions and improve long-term outcomes of real life interventions.

## Introduction

Translational research performed in different settings and populations confirmed findings of Randomized Control Trials (RCTs) that type 2 diabetes mellitus (DM2) prevention through lifestyle interventions can be effective and that results in terms of weight reduction and beneficial cardiovascular outcomes can be sustained in the long term [1–7]. However, results of weight reduction in translational research studies tend to be modest and the proportion of people who can maintain weight reduction is low compared with participants of randomized control trials [6]. There is scientific evidence that maintaining weight loss in the long term decreases the diabetes incidence by up to 89% among individuals at high risk of DM2 [8]. However, long-term weight loss maintenance is achieved only by around 20% of obese people who initially lost weight during lifestyle interventions [9].

Diabetes prevention in people at high risk is one of the most important challenges in primary health care to decrease the burden and complications of DM2. Therefore, improvement of the efficacy and effect duration in implementation programs is one of the biggest challenges to public health. Thus, there is a need to identify predictors of weight loss sustainability in DM2 prevention programs in primary health care patients.

The DE-PLAN project (Diabetes in Europe: Prevention Using Lifestyle, Physical Activity and Nutritional Intervention) was a translational research study aiming to assess the reach, adoption and implementation of the programme in diverse real life settings in 17 European countries. The aim of the DE-PLAN (initiated and sponsored by EU and local governments) was also to create a network of trained and experienced professionals to continue diabetes prevention across Europe [6,10–11].

The objective of this study was to identify factors predicting long-term successful weight reduction maintenance achieved during a DM2 prevention program in patients with high diabetes risk in primary health care.

## Materials and methods

### Research design and methods

This study was developed and evaluated as real life, primary healthcare setting implementation project. A detailed description of the methodology as well as one and three-year results are described elsewhere [6,7].

### Study population

The study sample consisted of patients from nine independent Primary Health Care practices in the city of Krakow, Poland. Participants aged over 25 years, with a high diabetes risk corresponding to a score >14 in the Finnish Diabetes Risk Score (FINDRISC) and without previously diagnosed diabetes and without chronic disease which could affect the results of the study were invited to participate in the study [6]. Out of 566 FINDRISC questionnaires completed, 368 respondents scored >14 and 275 agreed to undergo an oral glucose tolerance (OGTT) examination. Subsequently 262 were invited to participate in the lifestyle intervention program. All in all, 175 participants completed the one-year intervention and 113 of them completed the follow-up examination three years after the baseline examination. Nine people (eight with complete measurements) who participated in the three-year follow-up did not participate in the one year examination. Thus, information of all three measurements were available of 105 study participants who were included in the analysis [6].

This study followed the Good Clinical Practice guidelines and the guidelines of the Helsinki Declaration. The study protocol was approved by the Jagiellonian University Ethics Committee. The committee’s reference number is KBET/43/L/2006. All study participants gave their written informed consent prior to the participation in the study. The registration number for the clinical trial is ISRCTN (ID ISRCTN96692060).

### Description of intervention

The intervention followed the steps of the Diabetes Prevention Study (DPS) modified and adjusted to the local, primary health care setting [1,6–7, 10–11]. One individual and ten group sessions, focused on lifestyle changes were provided followed by six telephone sessions and two motivational letters over a period of 10 months. The lifestyle interventions were provided by primary health-care nurses, two from each health-care practice trained as prevention managers. The main intervention goals were weight loss, decrease of fat consumption, increase of fruit and vegetable consumption and increase in physical activity. Starting from week four of the intervention patients were offered free of charge participation in physical activity sessions twice a week. There were no other post-intervention contacts with the participants except follow-up measures at years 1 and 3 [6].

### Measurements, predictors and outcome variables

Patients were examined at baseline, after 12 and 36 months of the study. The examination procedure included: questionnaires (FINDRISC, baseline, clinical and lifestyle and quality of life) and biochemical tests including: fasting and 2-hour post-load glucose, serum triglycerides, HDL and total cholesterol. Impaired fasting glucose (IFG) was defined as fasting plasma glucose concentration of more than 6.0 and less than 7.0 mmol/l. Impaired glucose tolerance (IGT) was defined as glucose plasma concentration of more than 7.80 to less than 11.1 mmol/l after OGTT, diabetes mellitus (DM2) was defined as fasting glucose concentration from 7.0 mmol/l or from 11.1 mmol/l two hours after OGTT [6]. Body mass index (BMI) was calculated as weight (in light indoor clothes, kg) divided by height squared (m^2^), waist circumference was measured midway between the lowest rib and iliac crest, diastolic and systolic blood pressure were taken while sitting after 10 minutes rest.

Data regarding education, marital status, employment status, history of increased blood glucose, family history of diabetes, FINDRISC, smoking status, history of hypertension, history of depression were assessed by a self-reported questionnaire.

Lifestyle changes were measured both during examination after one year as well as during follow-up examination, three years after the baseline examination. Patients completed self-reported questionnaires regarding the consumption of vegetables and fruit, consumption of total fat and saturated fat, change of saturated fat to unsaturated, alcohol consumption and physical activity over the past year. Lifestyle goals’ achievement was defined as low if 1–3 goals were achieved and high if 4–5 goals were achieved.

Participants were categorized into two groups based on the weight change achieved at three year examination (two years after discontinuation of intervention): those who continued to lose weight or did not change the weight achieved one year after the initiation of the intervention (weight change ≤0; maintainers) and those who increased weight during follow up (weight change >0; non-maintainers).

### Statistical analyses

The data was analysed using STATISTICA version 12 (StatSoft, Inc. (2014), www.statsoft.com). The descriptive analyses are presented as percentages for categorical variables and means with standard deviations for continuous variables. Chi-square tests for categorical variables and t-tests for continuous ones were applied to compare the distribution between the potential predictors according to whether the participants maintained weight loss during follow up or not. Coefficients of contingency were calculated to assess correlations between the lifestyle variables (max. C for tables 2 x 2 = 0.707). Conditional stepwise logistic regression models were used to assess the association between the different predictors of weight loss maintenance. The covariates for the backward logistic regression model were chosen after revising scientific evidence on risk factors for type 2 diabetes and factors that may predict weight changes during the interventions. Even though age and sex were not statistically significantly associated with the outcome we kept them in the final model due to common practice. All the covariates were entered into the equation first. Variables were removed from the model according to the probability of the likelihood-ratio statistic based on conditional parameter estimates. The odds ratios (OR) and the respective 95% confidence intervals (CI) were calculated. A p-value of < 0,05 was considered as a level of statistical significance.

## Results

**Fig 1:**
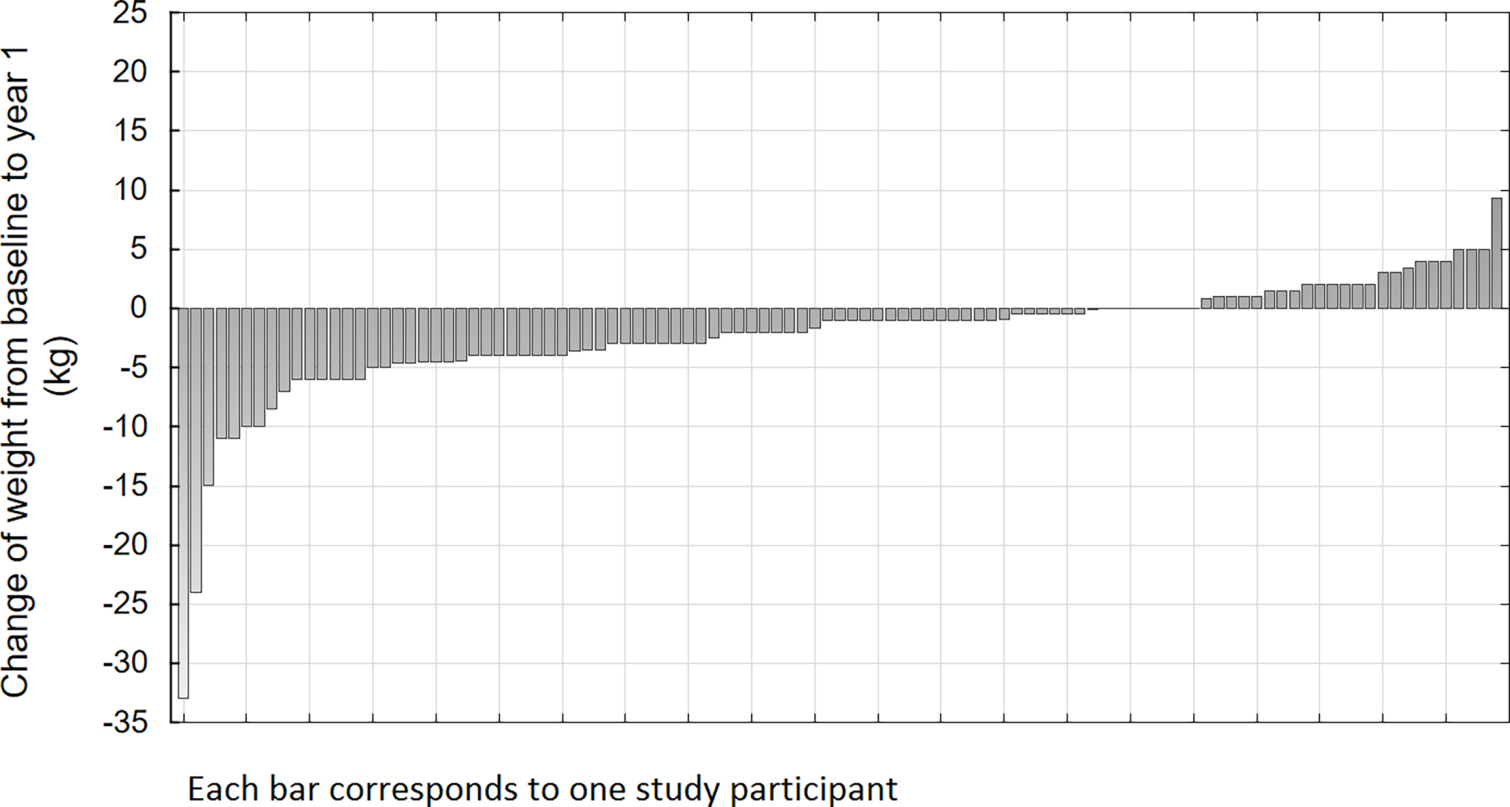
Waterfall charts of weight changes from baseline to year one of the study participants (n = 105).

**Fig 2:**
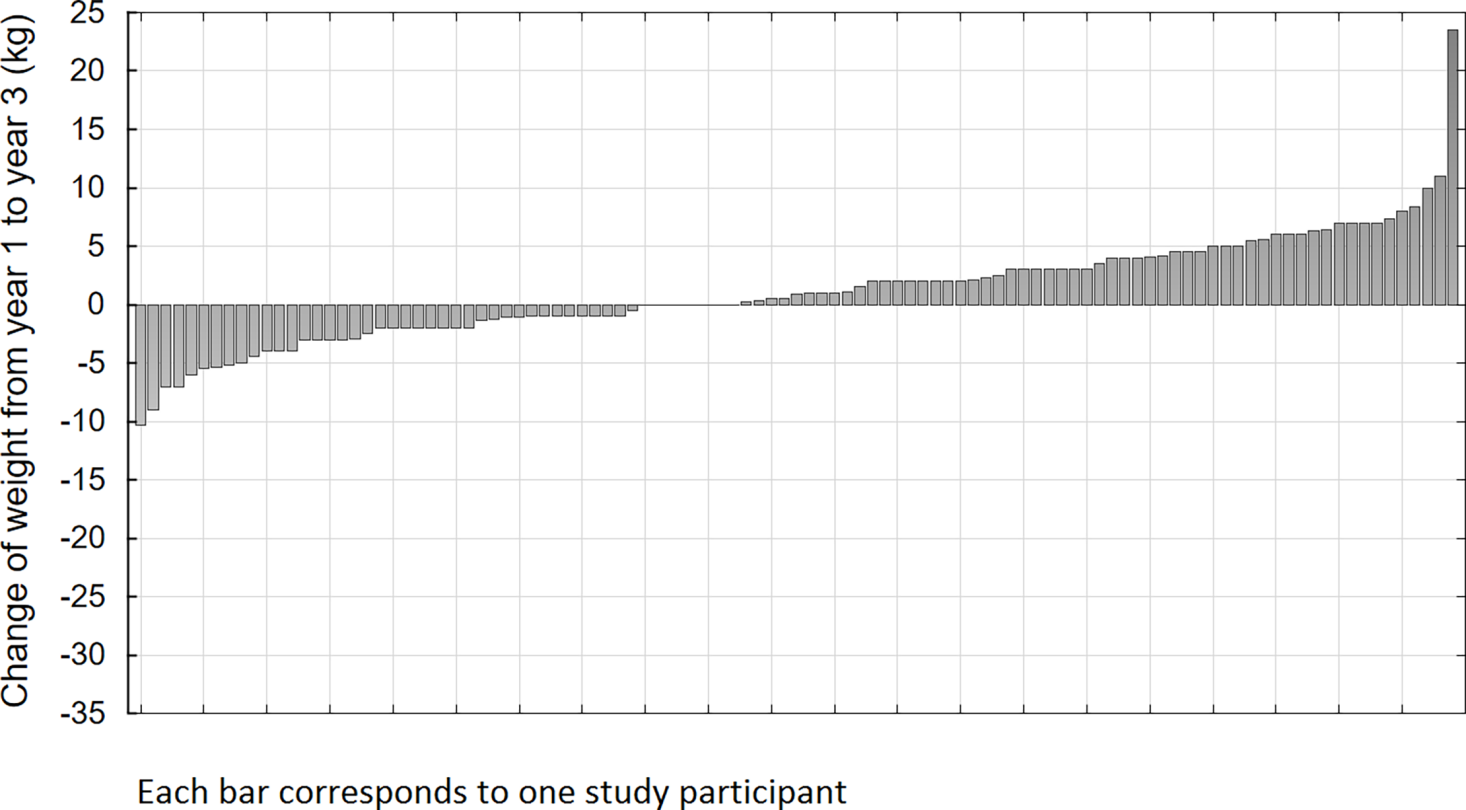
Waterfall charts of weight changes from year one to year three of the study participants (n = 105).

Out of 105 patients who completed all three examinations (baseline age 56.6 (standard deviation (SD) 10.7), BMI 31.1 kg/m^2^ (SD 4.9), FINDRISC 18.6 (SD 3.1), 13% of men) 73 patients (70%) successfully lost weight one year after initiation of the intervention (mean weight loss 4.2 kg, SD = 5.1) (Fig 1). Out of these 27 (37%) maintained weight loss during two year post-intervention follow-up (additional mean weight loss 2.1 kg, SD = 2.3) while 46 (63%) gained weight (mean weight increase 4.2 kg, SD = 3.7) (Fig 2).

Baseline clinical and metabolic characteristic of maintainers and non-maintainers as well as during one year and three years after the one-year intervention are presented in Table 1. The weight loss presented for year 1–3 years after the intervention corresponds to additional weight loss after the intervention has ended. Thus, the total weight loss achieved in the maintainers (27 of 73 study participants) two years after the intervention had finished was 6.54 kg (4.47 kg+2.0kg). The non-maintainers, on the other hand, returned to their initial weight at the start of the intervention (+0.21 kg). Reduction in waist circumference among maintainers was 5.26 cm and among no-maintainers 0.33 cm.

**Table 1:** Clinical and metabolic characteristic of people who maintained and did not maintain weight loss during follow-up.

	Baseline			Change from baseline to year 1			Change from year 1 to year 3		
	Maintainers	Non-maintainers	p value	Maintainers	Non-maintainers	p value	Maintainers	Non-maintainers	p value
	Mean (SD^1^)	Mean (SD)		Mean (SD)	Mean (SD)		Mean (SD)	Mean (SD)	
Age	58.0 (12.2)	54.7 (9.6)	0.203						
Weight (kg)	84.9 (14.4)	82.8 (15)	0.568	-4.47 (6.63)	-4.02 (3.97)	0.714	-2.07 (2.25)	4.23 (3.69)	<0.001
BMI^2^ (kg/m2)	31.2 (4.2)	31.3 (5.2)	0.916	-1.74 (2.36)	-1.47 (1.37)	0.548	-0.69 (0.93)	1.63 (1.44)	<0.001
WC^3^ (cm)	99.1 (9.9)	95.5 (11.6)	0.185	-5.93 (5.25)	-4.72 (5.12)	0.338	0.67 (6.02)	4.39 (4.39)	0.007
SBP^4^ (mmHg)	131.6 (13.4)	132 (14.9)	0.907	-1.65 (12.19)	-4.57 (18.27)	0.463	2.67 (17.58)	3.49 (16.28)	0.84
DBP^5^ (mmHg)	80.8 (7.1)	81.8 (9.7)	0.628	-1.09 (7.36)	-2.86 (9.48)	0.409	-0.41 (6.73)	1.58 (9.47)	0.343
Fasting glucose (mmol/l)	5.2 (0.6)	5.2 (0.8)	0.438	0.1 (0.52)	0.27 (0.74)	0.311	-0.05 (0.62)	-0.12 (0.91)	0.729
2-hour glucose (mmol/l)	5.5 (1.4)	5.8 (1.9)	0.946	0.37 (1.64)	0.07 (2.37)	0.56	0.11 (1.59)	0.34 (1.68)	0.566
TCH^6^ (mmol/l)	5.5 (1.0)	5.5 (0.8)	0.878	-0.17 (-0.95)	-0.18 (1.18)	0.977	0.02 (1.17)	-0.11 (1.12)	0.643
HDL^7^ (mmol/l)	1.4 (0.3)	1.4 (0.4)	0.733	-0.04 (0.32)	-0.04 (0.27)	0.982	-0.08 (0.31)	0.02 (0.65)	0.459
TG^8^ (mmol/l)	1.9 (1.4)	1.5 (0.7)	0.925	-0.31 (1.23)	0.08 (0.74)	0.091	0.03 (0.67)	-0.09 (0.71)	0.481
FINDRISC^9^	19.2 (2.6)	18.3 (3)	0.225	-3.41 (3.78)	-2.91 (3.41)	0.567	0.15 (2.93)\	0.7 (4.47)	0.572

^1^Standard deviation

^2^Body mass index

^3^Waist circumference

^4^Systolic blood pressure

^5^Diastolic blood pressure

^6^Total cholesterol

^7^High density lipoprotein

^8^Triglycerides

^9^Finnish Diabetes Risk Score

At baseline 15% of the weight loss maintainers and 24% of the non-maintainers had IFG or IGT (p = 0.483). Weight loss maintainers had more often baseline history of increased glucose (85% vs. 65%, p = 0.055). There were no other baseline differences between maintainers and non-maintainers. During follow-up five people developed diabetes, one of the maintainers and four among non-maintainers (p = 385), while IFG or IGT was present among 5 and 6 people in maintainers and non- maintainers respectively (p = 0.604)

**Fig 3:**
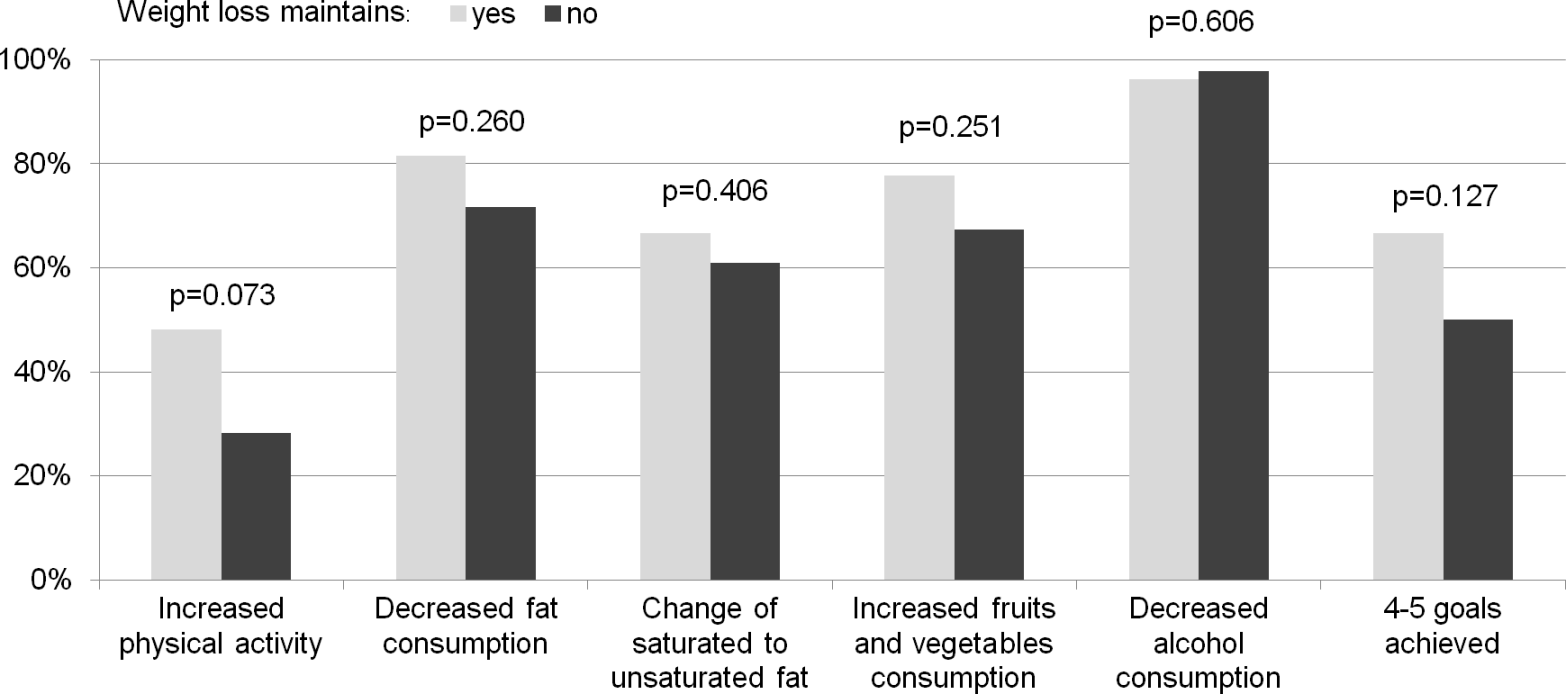
Lifestyle changes and lifestyle goals achieved after intervention (one year assessment).

**Fig 4:**
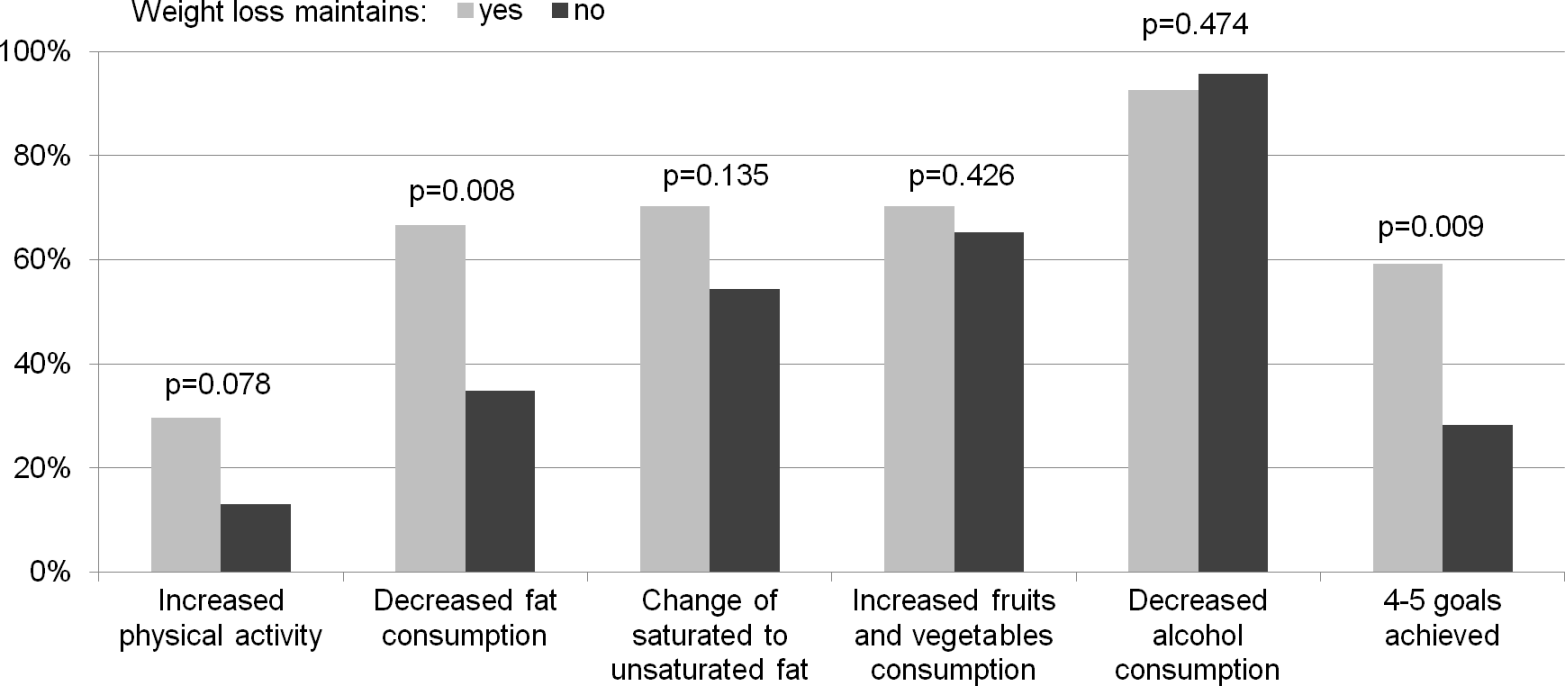
Lifestyle changes and lifestyle goals achieved during follow-up (three-year assessment).

During the intervention period people who maintained weight loss vs non-maintainers more often increased their physical activity (48% vs. 28%, p = 0.072). They also continued this lifestyle change during follow up period (30% vs 13%, p = 0.078) and more often continued to decrease fat in diet (67% vs 35%, p = 0.008) (Fig 3, Fig 4).

There were no differences in the number of achieved lifestyle goals after intervention but during follow-up period those who managed to maintain weight reduction more often achieved 4–5 lifestyle goals than those who did not (59% vs. 28%, p = 0.009) (Fig 3, Fig 4).

44% of weight loss maintainers and 67% of non-maintainers did not increase their physical activity both during intervention and during follow-up (p = 0.046), while 4% maintainers and 22% non-maintainers did not decrease total fat in diet in both observation periods (p = 0.035). In multivariable analysis baseline history of increased fasting glucose (OR = 4.53; 95% CI 1.13–18.15) was associated with weight loss maintenance. In the adjusted model, reduction of total fat in diet during follow-up was associated with a 4.3-fold increased odds of successful weight loss (95% CI 1.41–12.98) (Table 2).

**Table 2:** Unadjusted and adjusted odds ratios of predictors of weight loss maintenance during follow-up in the study participants.

	Unadjusted model		Adjusted model	
	OR^1^	95% CI^2^	OR	95% CI
Age	1.04	(0.97; 1.12)	1.03	(0.98; 1.09)
Sex (M/F)	4.61	(0.81; 26.31)	3.87	(0.81; 18.47)
Education (Basic and medium/High)	2.19	(0.32; 15.19)		
Marital status (Married or having a partner/Single or widow)	1.73	(0.41; 7.25)		
BMI^3^	1.05	(0.91; 1.22)		
Waist circumference				
History of Increased Glucose	4.47	(0.75; 26.51)	4.53	(1.13; 18.15)
Family History of Diabetes	1.49	(0.33; 6.79)		
History of Hypertension	1.19	(0.21; 6.71)		
Smoking currently	1.21	(0.23; 6.50)		
FINDRISC^4^	0.96	(0.73; 1.27)		
Increased physical activity over past year	2.18	(0.49; 9.60)		
Decreased consumption of fat over past year	5.99	(1.17; 30.58)	4.28	(1.41; 12.98)
Increased consumption of fruit and vegetables over past year	0.46	(0.09; 2.29)		
≥5% weight loss during intervention period	2.18	(0.49; 9.60)		

^1^Odds ratio

^2^Confidence interval

^3^Body mass index

^4^Finnish Diabetes Risk Score

Analysis of contingency coefficients confirmed that decrease of fat consumption during follow-up was strongly correlated with increase in consumption of vegetables and fruit C = 0.431 (max. C for tables 2 x 2 = 0.707).

## Discussion

Our study revealed that one third of the study participants achieved a total weight loss of 6.5 kg two-years after the intervention had ended. From a public health point of view, one of the mayor challenges is to identify factors that predicts successful weight maintenance on long-term. Despite scientific evidence of randomized clinical trials, that weight loss may be maintained [5–6,11–13], information on translating the results of RCT into the real world is still scant and evidence on long-term success rates of weight loss or maintenance is not sufficient. This study shows that weight maintenance can be achieved on long-term within the primary health setting and identified some predictors of successful weight loss maintenance.

We have reported earlier that weight reduction and favourable changes in cardiovascular risk factors, and decrease of DM2 risk achieved at one year were maintained at three-year follow-up [6]. In our study one year after initiation of intervention 23.4% of participants lost ≥5% weight (mean loss 7.9 kg, SD = 5.8) [7]. There is very scarce information about the long-term results of implementation studies, also little is known about the factors predicting long-term success [5–6,11–14]. Johnson et al suggested that while in RCTs effectiveness, also long-term, could be demonstrated as lowering of DM2 incidence, in real life prevention studies with usually small sample size weight reduction may have sufficient statistical power to be regarded as a marker of potential prevention long-term [13]. This is confirmed by the results of the Diabetes Prevention Program (DPP) where 55% reduction in T2DM incidence over 3 years’ follow-up was the result of a loss of 5 kg [15].

In the European Diabetes Prevention Study (EDIPS), also based on the experiences of the DPS, in the intervention group weight loss defined as ≥5% was achieved in 38% at year one, maintained among 28% at year 2 and among 23% at year 3 of the intervention. EDIPS participants who achieved weigh loss at year 1 had 64% lower DM2 incidence, those who maintained weight loss at year two and three had lower diabetes type 2 risk by 79% and 89% respectively [8]. Explanatory analysis of the EDIPS study suggests that sustained weight loss at year two and three of intervention enhanced the intervention effect [8].

Several definitions of successful weight loss maintenance have been proposed, however these usually referred to studies with largely obese individuals [9]. In general population there is a weight increase by 0.5 kg per year, thus, even a small weight reduction or weight maintenance should be considered as an important achievement in diabetes prevention [16]. In the DPP trial weight loss was the single most important factor in reducing diabetes incidence-for every kilogram of weight loss, diabetes incidence was reduced by 16 percent [15]. We have previously shown that maintenance of even modest weight reduction resulted in sustained improvement of clinical outcomes and diabetes risk [6]. Therefore, in our study (among high diabetes risk individuals but with moderate obesity) we decided to define maintainers as those who managed to continue to lose any weight during three-year follow-up or to maintain weight loss achieved during intervention (e.g. 2 years after the end of intervention). There are only a few studies investigating long-term results of lifestyle intervention on weight maintenance. In the National Weight Control Registry (NWCR) long-term weight loss maintenance is achieved only by around 20% of obese people who initially successfully lost weight during interventions [9].

People who maintained weight loss over long term were engaged in higher levels of activity, were on a low-calorie, low-fat diet, were eating breakfast regularly, and frequently were self-monitoring their weight [9]. Wing et Phelan suggest that people who manage to maintain weight loss continue to act like those who have just lost weight [9]. In the NWCR, people who did not manage to maintain weight loss in a long term reported deterioration of healthy behaviours: they significantly decreased their physical activity, increased percentage of fat in diet and decreased dietary restraints [9].

In our study history of increased glucose reported at baseline (original question was “Have you ever been told by any medical professional that you had increased glucose”) was an independent predictor of long-term weight loss maintenance. This is in line with the results of the NWCR where “medical triggers” were the most common factors leading participants to successful weight loss [9]. In our study also meeting ≥5% weight loss one year after the initiation of intervention was most effective in people with a history of increased glucose [7].

Information regarding history of increased glucose, family history of diabetes as well as baseline anthropometric and biochemical results were summarized by prevention manager during the first individual session and during the following first group session and used to explain the individual diabetes risk as a motivation tool to convince patients to change their behaviour [6,7]. We suggest that medical advice and awareness of the risk of disease are essential to achieve long-term weight loss.

Similarly to the NWCR and other studies, decreased consumption of fat in diet during follow-up period was a strong independent factor of weight loss maintenance [9,2]. In the DPS, 4 years after discontinuation of intervention people who were intervened continued to reduce total energy and percentage of fat in diet, consumption of saturated fat, monounsaturated fat, trans fatty acids and alcohol [2]. However, the total hours of physical activity did not differ between the intervened vs. non-intervened [2]. In our study people who managed to maintain weight loss over long term vs. non-maintainers did not differ in lifestyle achievements during intervention period, except for more often improved physical activity. It is not surprising as all of them were those who had successfully managed to lose weight during intervention. However, during follow-up those who maintained weight loss over long term more often continued to increase physical activity and to decrease fat in diet. Also, more maintainers than non-maintainers achieved 4–5 lifestyle changes during follow-up. This suggests that a continued adherence to life changes during post-intervention period is very important to achieve long-term weight loss maintenance.

We revealed interesting information when we checked the number of people who did not change physical activity and total fat consumption both during intervention period and follow-up. Among non-maintainers as much as 2/3 had not increased physical activity during both periods while 1/5 had not decreased total fat in diet. These data might suggest that for people who did not manage to sustain weight reduction it was easier to change diet than to increase physical activity. According to Santos et al, while physical activity had a modest impact during the weight loss period, it was essential to weight loss maintenance [17]. In the NWCR, keeping stable weight over a long term period was associated with a high level of physical activity of approximately 1 hour per day [9]. Our results suggest that while designing future interventions more emphasis should be placed on physical activity changes. The importance of lifestyle modifications on long-term results was shown in the 10 years’ follow-up observation in the DPP. There was a lower cumulative diabetes incidence in the lifestyle group vs. placebo and metformin group. This effect prevailed even in the lifestyle group which partly regained weight. This result shows that the effects of lifestyle intervention might be beneficial even if the weight is partly regained [4].

In the published efficacy trials lifestyle interventions usually lasted several years [1–4]. In the DPS study there were 20 sessions during 3-year intervention [1–3] while in our study, during 10 months, there were 11 counselling sessions followed by 6 motivational telephone calls and 2 letters. In the meta-analysis of the real-world diabetes prevention studies published by Aziz et al. it was shown that intensity and duration of intervention in RCTs is usually incomparable to real-life implementation studies [12]. Aziz found, confirming earlier research, that lifestyle-focused diabetes prevention programs that include a ‘high’ degree of contact and long duration are more likely to achieve effective outcomes, especially in terms of weight loss [12–14]. In the meta-analysis by Montessi et al several strategies to promote sustained weight loss have been evaluated: group sessions delivered twice a month for 1 year after the weight loss phase and keeping patients in active treatment to help maintain weight loss [17]. There is also evidence that long-term self-help group pressure and support might help some people maintain weight [18]. According to Montessi et al and others the extended intervention with frequent face to face, telephone or internet contacts provides support and motivation needed to continue weight control behaviours [18–21].

Usually in implementation studies there are much lower and realistic resources given for intervention which also need to be adapted to local, cultural and health care possibilities [11–14]. Consequently, as seen also in our study, the achieved weight reduction usually is modest when compared with RCTs [5–7,11–14]. However, even with such limited resources, it is necessary to redesign interventions for longer duration with sufficient maintenance phase adapted to local and individual possibilities (in person, telephone, internet/app contacts as well as self-help support groups) in order to improve the efficacy and cost-efficiency of future prevention programs [18–22].

Some strengths and limitations of our study need to be discussed. This is one of the first studies investigating the predictors of weight loss maintenance in real-life, real-setting implementation of DM2 prevention intervention. The participants in our study were volunteers and, like many other studies, the study predominantly attracted women [6,7, 22]. We have currently published the results of the analyses showing that men, those who work and those with a worse health profile, are less likely to participate and complete interventions [22]. We suggested that in real-life diabetes prevention interventions targeted strategies are needed to improve male participation and to reach those who are working as well as people with a higher risk profile [22]. In the weight loss maintenance there are very important psychological and behavioural factors which were not examined in our study [9,23–24]. In the light of a very poor male participation and success these factors should be also further investigated for both sexes. However, even though low, the percentage of the weight loss maintainers, is similar to already published studies on weight loss maintenance [9]. Finally, our study is a retrospective one-arm association study with a small number of study participants divided into two groups based on weight reduction maintenance. Thus, the power to investigate predictors of successful weight maintenance may be low. However, these results may be used as exploratory findings guiding future research in bigger population samples of translational research identifying possible predictors of weight maintenance on long-term.

There is also growing evidence that the interventions should be individualised and as suggested by Hjorth et al [25] the type of diet should be offered in regard to the glucose/insulin status of participants.

In conclusion, a deepened insight into participant characteristics, including psychological and behavioural factors, which independently predict weight loss maintenance, is critical for further work of health care providers in real life diabetes prevention initiatives to identify those who are most likely to succeed. Novel strategies and designs including extended motivation phase and focus on physical activity modification and individualized intervention should be implemented to improve long-term results of DM2 interventions. Finally, this study provides valuable insight of parts of translational research in type 2 diabetes prevention and that weight changes can be maintained and may have important implications for implementation and scalability of diabetes prevention in Poland and Europe in general.

## Supporting information

S1 File DEPLAN data used for the analyses presented in this paper. (XLSX)

S2 File DEPLAN data list of variables used for the analyses. (DOCX)
