# Supplementary material for: Predictors of long term weight loss maintenance in patients at high risk of type 2 diabetes participating in a lifestyle intervention program in primary health care: The DE-PLAN study
Source: PLoS One. 2018 Mar 23;13(3):e0194589. doi: 10.1371/journal.pone.0194589 (PMC5865727; doi:10.1371/journal.pone.0194589)
Supplement: S2 File — (DOCX) [file pone.0194589.s002.docx]

Variables

|  | 1AGE |
| --- | --- |
|  | 1SEX |
|  | 1BQ1Marital Stat |
|  | 1BQ2Education |
|  | 1Working status |
|  | 1BQ4Diabetes hist |
|  | 1BQ5Diabetes hist fath |
|  | 1BQ6Diabetes hist moth |
|  | 1BQ7Siblings number |
|  | 1BQ8Diabetes hist sibl |
|  | 1BQ9_01Diseases HT |
|  | 1BQ9_02Diseases CInsuff |
|  | 1BQ9_03Diseases MIC |
|  | 1BQ9_04Diseases MIC |
|  | 1BQ9_05Diseases infarct |
|  | 1BQ9_06Diseases by-pass |
|  | 1BQ9_07Diseases |
|  | 1BQ9_08Diseases |
|  | 1BQ9_09Diseases cholest |
|  | 1BQ9_10Diseases depression |
|  | 1BQ9_11Diseases inability |
|  | 1BQ9_12Diseases other chronic |
|  | 1BQ12smoking ever |
|  | 1BQ13smoking |
|  | 1BQ14smoking |
|  | 1BQ15_01smoking |
|  | 1BQ15_02smoking |
|  | 1BQ15_03smoking |
|  | 1BQ16smoking Have you planned to quit |
|  | 1BQ17ph_act How demanding is your work… |
|  | 1BQ18ph_act |
|  | 1BQ19ph_act |
|  | 1BQ20ph_act |
|  | 1BQ21ph_act |
|  | 1BQ22ph_act |
|  | 1BQ23ph_act |
|  | 1BQ24ph_act |
|  | 1BQ25ph_act have you increased physical act |
|  | 1BQ26food |
|  | 1BQ27_01food |
|  | 1BQ27_02food |
|  | 1BQ27_03food |
|  | 1BQ27_04food |
|  | 1BQ27_05food |
|  | 1BQ28food |
|  | 1BQ29food |
|  | 1BQ30food |
|  | 1BQ31food |
|  | 1BQ32food |
|  | 1BQ33_01food |
|  | 1BQ33_02food |
|  | 1BQ33_03food |
|  | 1BQ33_04food |
|  | 1BQ33_05food |
|  | 1BQ34_01food |
|  | 1BQ34_02food |
|  | 1BQ34_03food |
|  | 1BQ34_04food |
|  | 1BQ34_05food |
|  | 1BQ34_06food |
|  | 1BQ34_07food |
|  | 1BQ34_08food |
|  | 1BQ34_09food |
|  | 1BQ34_10food |
|  | 1BQ35food |
|  | 1BQ36food |
|  | 1BQ37_01food |
|  | 1BQ37_02food |
|  | 1BQ37_03food |
|  | 1BQ37_04food |
|  | 1BQ38_01food |
|  | 1BQ38_02food |
|  | 1BQ38_03food |
|  | 1BQ38_04food |
|  | 1BQ39 |
|  | 1BQ40 |
|  | 1BQ41_01food |
|  | 1BQ41_02food |
|  | 1BQ41_03food |
|  | 1BQ41_04food |
|  | 1BQ41_05food |
|  | 1BQ41_06food |
|  | 1BQ41_07food |
|  | 1BQ41_08food |
|  | 1BQ41_09food |
|  | 1BQ41_10food |
|  | 1BQ41_11food |
|  | 1BQ41_12food |
|  | 1BQ42food FAT |
|  | 1BQ43sat FAT |
|  | 1BQ44 vegetables |
|  | 1BQ45 alcohol |
|  | 1BQ46 lost weight |
|  | 2AGE |
|  | 2BQ1Marital Stat |
|  | 2BQ2Education |
|  | 2Working status |
|  | 2BQ4Diabetes hist |
|  | 2BQ5Diabetes hist fath |
|  | 2BQ6Diabetes hist moth |
|  | 2BQ7Siblings number |
|  | 2BQ8Diabetes hist sibl |
|  | 2BQ9_01Diseases HT |
|  | 2BQ9_02Diseases CInsuff |
|  | 2BQ9_03Diseases MIC |
|  | 2BQ9_04Diseases MIC |
|  | 2BQ9_05Diseases infarct |
|  | 2BQ9_06Diseases by-pass |
|  | 2BQ9_07Diseases |
|  | 2BQ9_08Diseases |
|  | 2BQ9_09Diseases cholest |
|  | 2BQ9_10Diseases depression |
|  | 2BQ9_11Diseases inability |
|  | 2BQ9_12Diseases other chronic |
|  | 2BQ12smoking ever |
|  | 2BQ13smoking |
|  | 2BQ14smoking |
|  | 2BQ15_01smoking |
|  | 2BQ15_02smoking |
|  | 2BQ15_03smoking |
|  | 2BQ16smoking Have you planned to quit |
|  | 2BQ17ph_act How demanding is your work… |
|  | 2BQ18ph_act |
|  | 2BQ19ph_act |
|  | 2BQ20ph_act |
|  | 2BQ21ph_act |
|  | 2BQ22ph_act |
|  | 2BQ23ph_act |
|  | 2BQ24ph_act |
|  | 2BQ25ph_act have you increased physical act |
|  | 2BQ26food |
|  | 2BQ27_01food |
|  | 2BQ27_02food |
|  | 2BQ27_03food |
|  | 2BQ27_04food |
|  | 2BQ27_05food |
|  | 2BQ28food |
|  | 2BQ29food |
|  | 2BQ30food |
|  | 2BQ31food |
|  | 2BQ32food |
|  | 2BQ33_01food |
|  | 2BQ33_02food |
|  | 2BQ33_03food |
|  | 2BQ33_04food |
|  | 2BQ33_05food |
|  | 2BQ34_01food |
|  | 2BQ34_02food |
|  | 2BQ34_03food |
|  | 2BQ34_04food |
|  | 2BQ34_05food |
|  | 2BQ34_06food |
|  | 2BQ34_07food |
|  | 2BQ34_08food |
|  | 2BQ34_09food |
|  | 2BQ34_10food |
|  | 2BQ35food |
|  | 2BQ36food |
|  | 2BQ37_01food |
|  | 2BQ37_02food |
|  | 2BQ37_03food |
|  | 2BQ37_04food |
|  | 2BQ38_01food |
|  | 2BQ38_02food |
|  | 2BQ38_03food |
|  | 2BQ38_04food |
|  | 2BQ39 |
|  | 2BQ40 |
|  | 2BQ41_01food |
|  | 2BQ41_02food |
|  | 2BQ41_03food |
|  | 2BQ41_04food |
|  | 2BQ41_05food |
|  | 2BQ41_06food |
|  | 2BQ41_07food |
|  | 2BQ41_08food |
|  | 2BQ41_09food |
|  | 2BQ41_10food |
|  | 2BQ41_11food |
|  | 2BQ41_12food |
|  | 2BQ42food FAT |
|  | 2BQ43sat FAT |
|  | 2BQ44 vegetables |
|  | 2BQ45 alcohol |
|  | 2BQ46 lost weight |
|  | 3AGE |
|  | 3BQ1Marital Stat |
|  | 3BQ2Education |
|  | 3Working status |
|  | 3BQ4Diabetes hist |
|  | 3BQ5Diabetes hist fath |
|  | 3BQ6Diabetes hist moth |
|  | 3BQ7Siblings number |
|  | 3BQ8Diabetes hist sibl |
|  | 3BQ9_01Diseases HT |
|  | 3BQ9_02Diseases CInsuff |
|  | 3BQ9_03Diseases MIC |
|  | 3BQ9_04Diseases MIC |
|  | 3BQ9_05Diseases infarct |
|  | 3BQ9_06Diseases by-pass |
|  | 3BQ9_07Diseases |
|  | 3BQ9_08Diseases |
|  | 3BQ9_09Diseases cholest |
|  | 3BQ9_10Diseases depression |
|  | 3BQ9_11Diseases inability |
|  | 3BQ9_12Diseases other chronic |
|  | 3BQ12smoking ever |
|  | 3BQ13smoking |
|  | 3BQ14smoking |
|  | 3BQ15_01smoking |
|  | 3BQ15_02smoking |
|  | 3BQ15_03smoking |
|  | 3BQ16smoking Have you planned to quit |
|  | 3BQ17ph_act How demanding is your work… |
|  | 3BQ18ph_act |
|  | 3BQ19ph_act |
|  | 3BQ20ph_act |
|  | 3BQ21ph_act |
|  | 3BQ22ph_act |
|  | 3BQ23ph_act |
|  | 3BQ24ph_act |
|  | 3BQ25ph_act have you increased physical act |
|  | 3BQ26food |
|  | 3BQ27_01food |
|  | 3BQ27_02food |
|  | 3BQ27_03food |
|  | 3BQ27_04food |
|  | 3BQ27_05food |
|  | 3BQ28food |
|  | 3BQ29food |
|  | 3BQ30food |
|  | 3BQ31food |
|  | 3BQ32food |
|  | 3BQ33_01food |
|  | 3BQ33_02food |
|  | 3BQ33_03food |
|  | 3BQ33_04food |
|  | 3BQ33_05food |
|  | 3BQ34_01food |
|  | 3BQ34_02food |
|  | 3BQ34_03food |
|  | 3BQ34_04food |
|  | 3BQ34_05food |
|  | 3BQ34_06food |
|  | 3BQ34_07food |
|  | 3BQ34_08food |
|  | 3BQ34_09food |
|  | 3BQ34_10food |
|  | 3BQ35food |
|  | 3BQ36food |
|  | 3BQ37_01food |
|  | 3BQ37_02food |
|  | 3BQ37_03food |
|  | 3BQ37_04food |
|  | 3BQ38_01food |
|  | 3BQ38_02food |
|  | 3BQ38_03food |
|  | 3BQ38_04food |
|  | 3BQ39 |
|  | 3BQ40 |
|  | 3BQ41_01food |
|  | 3BQ41_02food |
|  | 3BQ41_03food |
|  | 3BQ41_04food |
|  | 3BQ41_05food |
|  | 3BQ41_06food |
|  | 3BQ41_07food |
|  | 3BQ41_08food |
|  | 3BQ41_09food |
|  | 3BQ41_10food |
|  | 3BQ41_11food |
|  | 3BQ41_12food |
|  | 3BQ42food FAT |
|  | 3BQ43sat FAT |
|  | 3BQ44 vegetables |
|  | 3BQ45 alcohol |
|  | 3BQ46 lost weight |
|  | 1FRS01 age |
|  | 1FRS02 bmi |
|  | 1FRS03 waist |
|  | 1FRS04ph_act |
|  | 1FRS05 veg |
|  | 1FRS06 HT |
|  | 1FRS07 Hyperglic |
|  | 1FRS08 Fam hist diab |
|  | 1FRS09 Sum |
|  | 1FRS10 Risk |
|  | 2FRS01 age |
|  | 2FRS02 bmi |
|  | 2FRS03 waist |
|  | 2FRS04ph_act |
|  | 2FRS05 veg |
|  | 2FRS06 HT |
|  | 2FRS07 Hyperglic |
|  | 2FRS08 Fam hist diab |
|  | 2FRS09 Sum |
|  | 2FRS10 Risk |
|  | 3FRS01 age |
|  | 3FRS02 bmi |
|  | 3FRS03 waist |
|  | 3FRS04ph_act |
|  | 3FRS05 veg |
|  | 3FRS06 HT |
|  | 3FRS07 Hyperglic |
|  | 3FRS08 Fam hist diab |
|  | 3FRS09 Sum |
|  | 3FRS10 Risk |
|  | 1CDCF01 FRS Sum |
|  | 1CDCF03_01 Dis |
|  | 1CDCF03_02 Dis |
|  | 1CDCF03_03 Dis |
|  | 1CDCF03_04 Dis |
|  | 1CDCF03_05 Dis |
|  | 1CDCF03_06 Dis |
|  | 1CDCF03_07 Dis |
|  | 1CDCF04 height |
|  | 1CDCF05 weight |
|  | 1CDCF07 waist |
|  | 1CDCF08_01 SBP1 |
|  | 1CDCF08_01 DBP1 |
|  | 1CDCF08_02 SBP2 |
|  | 1CDCF08_02 DBP2 |
|  | 1CDCF11_02 plasma/serum |
|  | 1CDCF11_03 |
|  | 1CDCF11_04 Fasting glucose |
|  | 1CDCF11_05 OGTT glucose |
|  | 1CDCF11_06 |
|  | 1CDCF12_03 plasma/serum |
|  | 1CDCF12_04 overnight fast |
|  | 1CDCF12_05 TCH |
|  | 1CDCF12_06 HDL |
|  | 1CDCF12_07 TRI |
|  | 2CDCF01 FRS Sum |
|  | 2CDCF03_01 Dis |
|  | 2CDCF03_02 Dis |
|  | 2CDCF03_03 Dis |
|  | 2CDCF03_04 Dis |
|  | 2CDCF03_05 Dis |
|  | 2CDCF03_06 Dis |
|  | 2CDCF03_07 Dis |
|  | 2CDCF04 height |
|  | 2CDCF05 weight |
|  | 2CDCF07 waist |
|  | 2CDCF08_01 SBP1 |
|  | 2CDCF08_01 DBP1 |
|  | 2CDCF08_02 SBP2 |
|  | 2CDCF08_02 DBP2 |
|  | 2CDCF11_02 plasma/serum |
|  | 2CDCF11_03 |
|  | 2CDCF11_04 Fasting glucose |
|  | 2CDCF11_05 OGTT glucose |
|  | 2CDCF11_06 |
|  | 2CDCF12_03 plasma/serum |
|  | 2CDCF12_04 overnight fast |
|  | 2CDCF12_05 TCH |
|  | 2CDCF12_06 HDL |
|  | 2CDCF12_07 TRI |
|  | 3CDCF01 FRS Sum |
|  | 3CDCF03_01 Dis |
|  | 3CDCF03_02 Dis |
|  | 3CDCF03_03 Dis |
|  | 3CDCF03_04 Dis |
|  | 3CDCF03_05 Dis |
|  | 3CDCF03_06 Dis |
|  | 3CDCF03_07 Dis |
|  | 3CDCF04 height |
|  | 3CDCF05 weight |
|  | 3CDCF07 waist |
|  | 3CDCF08_01 SBP1 |
|  | 3CDCF08_01 DBP1 |
|  | 3CDCF08_02 SBP2 |
|  | 3CDCF08_02 DBP2 |
|  | 3CDCF11_02 plasma/serum |
|  | 3CDCF11_03 |
|  | 3CDCF11_04 Fasting glucose |
|  | 3CDCF11_05 OGTT glucose |
|  | 3CDCF11_06 |
|  | 3CDCF12_03 plasma/serum |
|  | 3CDCF12_04 overnight fast |
|  | 3CDCF12_05 TCH |
|  | 3CDCF12_06 HDL |
|  | 3CDCF12_07 TRI |
